# TNIP1 Regulates *Cutibacterium acnes*-Induced Innate Immune Functions in Epidermal Keratinocytes

**DOI:** 10.3389/fimmu.2018.02155

**Published:** 2018-09-24

**Authors:** Lilla Erdei, Beáta Szilvia Bolla, Renáta Bozó, Gábor Tax, Edit Urbán, Lajos Kemény, Kornélia Szabó

**Affiliations:** ^1^Department of Dermatology and Allergology, University of Szeged, Szeged, Hungary; ^2^Institute of Clinical Microbiology, University of Szeged, Szeged, Hungary; ^3^MTA-SZTE Dermatological Research Group, Szeged, Hungary

**Keywords:** TNIP1, TLR signaling, negative regulator, epidermal keratinocytes, cutibacterium acnes

## Abstract

Human skin cells recognize the presence of the skin microbiome through pathogen recognition receptors. Epidermal keratinocytes are known to activate toll-like receptors (TLRs) 2 and 4 in response to the commensal *Cutibacterium acnes* (*C. acnes*, formerly known as *Propionibacterium acnes*) bacterium and subsequently to induce innate immune and inflammatory events. These events may lead to the appearance of macroscopic inflammatory acne lesions in puberty: comedos, papules and, pustules. Healthy skin does not exhibit inflammation or skin lesions, even in the continuous presence of the same microbes. As the molecular mechanism for this duality is still unclear, we aimed to identify factors and mechanisms that control the innate immune response to *C. acnes* in keratinocytes using a human immortalized keratinocyte cell line, HPV-KER, normal human keratinocytes (NHEK) and an organotypic skin model (OSM). TNIP1, a negative regulator of the NF-κB signaling pathway, was found to be expressed in HPV-KER cells, and its expression was rapidly induced in response to *C. acnes* treatment, which was confirmed in NHEK cells and OSMs. Expression changes were not dependent on the *C. acnes* strain. However, we found that the extent of expression was dependent on *C. acnes* dose. Bacterial-induced changes in TNIP1 expression were regulated by signaling pathways involving NF-κB, p38, MAPKK and JNK. Experimental modification of TNIP1 levels affected constitutive and *C. acnes*-induced NF-κB promoter activities and subsequent inflammatory cytokine and chemokine mRNA and protein levels. These results suggest an important role for this negative regulator in the control of bacterially induced TLR signaling pathways in keratinocytes. We showed that all-trans retinoic acid (ATRA) induced elevated TNIP1 expression in HPV-KER cells and also in OSMs, where TNIP1 levels increased throughout the epidermis. ATRA also reduced constitutive and bacterium-induced levels of TNFα, CCL5 and TLR2, while simultaneously increasing CXCL8 and TLR4 expression. Based on these findings, we propose that ATRA may exhibit dual effects in acne therapy by both affecting the expression of the negative regulator TNIP1 and attenuating TLR2-induced inflammation. Overall, TNIP1, as a possible regulator of *C. acnes*-induced innate immune and inflammatory events in keratinocytes, may play important roles in the maintenance of epidermal homeostasis.

## Introduction

The human skin harbors a specialized microbiome, which plays an important role in the regulation and maintenance of epidermal homeostasis. Various members of the microbiome are also important in the pathogenesis of several skin diseases, most of which lead to exaggerated innate immune and inflammatory events. Examples of pathogenesis involving bacteria present in the microbiome include *Malassezia spp* in seborrheic dermatitis, *Staphylococcus aureus* in atopic dermatitis and *Cutibacterium acnes* (*C. acnes*, formerly known as *Propionibacterium acnes*) in acne vulgaris (1–4).

*C. acnes* becomes a dominant species in the pilosebaceous units during puberty by colonizing the hair follicle region, often forming a biofilm (5, 6). Human epidermal keratinocytes sense the presence of this bacterium with pathogen recognition receptors. *C. acnes* recognition is mostly mediated by the activation of toll-like receptors (TLRs) 2 and 4, induction of the canonical signaling pathway and subsequent innate immune and inflammatory events (7, 8). An important mediator of this cascade is the NF-κB transcription factor, which regulates the expression of key genes in the initialization and maintenance of downstream responses. These genes include different cytokines, such as tumor necrosis factor α (TNFα), interleukin (IL) 1α, IL1β, and IL6, chemokines, such as C-X-C motif chemokine ligand 8 (CXCL8) and C-C motif chemokine ligand 5 (CCL5), antibacterial peptides, such as human beta defensin 2 (hBD2), and other inflammatory mediators (9, 10). The innate immune activation of keratinocytes and the inflammatory milieu they generate in their environment favors the activation of other cell types, including sebocytes, dendritic cells and macrophages. Adaptive immune events are also induced, leading to the activation of the Th1/Th17 pathway (11, 12). Overall, these events contribute to the induction of the characteristic inflammatory symptoms during disease pathogenesis in adolescents. Inflammation and acne lesions are generally present transiently. In adolescents the inflamed follicles heal by themselves and the affected individuals often do not exhibit any residual signs. In the early twenties, even after resolution of the disease, *C. acnes* still dominates the microflora, especially in the sebum-rich skin regions. However, the bacterium does not usually provoke immune activation and inflammation in keratinocytes and/or other immune cells. This age-dependent response to *C. acnes* indicates different mechanisms controlling the downstream events induced by the bacterium.

Several negative regulators of the TLR and NF-κB signaling pathways have been identified in the past decade (13, 14). Through their regulatory functions, these negative regulators play important roles in the protection against extreme and prolonged activation of TLR and NF-κB downstream elements, both of which may lead to uncontrolled inflammation and tissue damage. TNIP1 (TNFAIP3 interacting protein 1), one such negative regulator, was identified as an interacting partner of TNFAIP3 (TNF alpha induced protein 3). TNIP1 has been shown to affect different signaling pathways by interacting directly with various proteins, including NEMO, TRAF1, p105, FADD, and RIP1. As a consequence, the transcription factor NF-κB and the TLR-MYD88 signaling cascade is inhibited and apoptotic and autoimmune events are negatively affected (15, 16). TNIP1 is known to be expressed in keratinocytes, where this protein controls cell proliferation partly due to the regulation of ERK1/2 signaling (17). TNIP1 promoter activity may be regulated by NF-κB, PPAR, retinoic acid receptors and also SP sites. These factors contribute to its basal and inducible activation possibly in a cell-type-specific manner (18–20). TNIP1's role in the regulation of TLR signaling in keratinocytes has been suggested in a recent study showing that its attenuation sensitizes HaCaT keratinocytes to synthetic TLR ligand treatment (21).

Currently, it is not clear whether negative regulators of the TLR and NF-κB signaling pathways play a role in the regulation of human microbiome-induced signaling events. Thus, we aimed to analyse the possible role of TNIP1 in *C. acnes*-induced innate immune and inflammatory events using *in vitro* cultured immortalized human keratinocytes and organotypic skin models (OSMs).

Our results show that, upon *C. acnes* treatment, TNIP1 expression is rapidly induced in a dose-dependent manner, and this induction is regulated by the NF-κB and JNK pathways and to some extent by p38- and MAPKK-dependent signaling pathways. In addition, modified TNIP1 levels affect the outcome of bacterium-induced molecular events, suggesting that this molecule acts through a negative regulatory feedback loop.

Retinoids, including all-trans retinoic acid (ATRA), are used as effective acne drugs and act by promoting cell proliferation, inhibiting keratinocyte terminal differentiation, decreasing sebum production and, as a result, indirectly reducing the amount of *C. acnes* (22). We aimed to investigate whether ATRA also affects the innate immune function of keratinocytes and found that ATRA treatment increases TNIP1 expression levels in our model systems. Our results reveal a possible mode of retinoid action that has not been explored previously.

## Materials and methods

### Cell cultures and models

The human immortalized keratinocyte cell line HPV-KER (23) and normal human epidermal keratinocytes (NHEK) were used for our experiments. Both cell types were cultured in keratinocyte serum-free medium (KSFM, Life Technologies, Carlsbad, United States) containing 1% antibiotic/antimycotic (AB/AM, Sigma Aldrich, St. Louis, MO, United States) solution and supplemented with epidermal growth factor and brain pituitary extract under standard laboratory conditions (37°C in a humidified atmosphere containing 5% v/v CO_2_).

NHEKs and *ex vivo* skin biopsies were obtained from skin specimens collected from the Plastic Surgery Unit of our department. Written informed consent was obtained from investigated individuals. The study was approved by the Human Investigation Review Board of the University of Szeged (PSO-EDAFN-002, 23 February 2015, Szeged, Hungary) and complying with the ethical standards in accordance with the Helsinki Declaration. NHEK cells were isolated from the skin samples as described earlier (24). For *ex vivo* organotypic skin models (OSMs), 6 mm punch biopsies were washed first with normal saline solution (NSS) containing 2% AB/AM, followed by a wash in AB/AM-free NSS. Subsequently, they were placed onto the upper chambers of Transwell® cell culture inserts (Corning, New York, United States) and kept at the air-liquid interphase. This way, the dermal part of the biopsies were in contact with DMEM F12 liquid culture medium (Lonza, Basel, Switzerland) supplemented with 10% fetal bovine serum (EuroClone, Milan, Italy) lacking AB/AM.

### Treatments

For bacterial treatment, HPV-KER and NHEK cells were plated in AB/AM-free KSFM culture medium and co-cultured with live *C. acnes* strains belonging to different phylogenetic groups within the species (889, ATCC 11828, 6609) at various multiplicity of infection (MOI). *Ex vivo* skin models were treated with the *C. acnes 889* strain at a density of 3 × 10^7^ cfu/cm^2^ for 24 h. *C. acnes* strains were cultured and stored as previously described in detail (10).

To analyze the effect of the active form of retinoic acid, ATRA was dissolved in DMSO and a 10^−6^ M concentration was applied to HPV-KER cells for 48 h before *C. acnes* challenge. ATRA was applied to OSMs at a 1.5 × 10^−6^ M concentration for 24 h. As a control, cells were subjected to DMSO treatment without the active ingredient.

The selective inhibitors of JNK (sp 600125, 10 μM), NF-κB (Bay 11-7085, 10 μM), p38 (sb 203580, 10 μM), MAPKK (PD 098059, 20 μM), STAT1 (Fludarabine, 25 μM) and STAT3 (Stattic, 5 μM), or, as a control, DMSO were applied to the cells for 1 h (all reagents from Sigma Aldrich, St. Louis, MO, United States).

### Transfection, plasmids and siRNA-mediated gene silencing

Transient transfection experiments were performed using the X-tremeGENE 9 DNA Transfection Reagent (Roche, Indiana, United States). For the overexpression studies, HPV-KER cells were plated in 12-well plates (100,000 cells/well), transfected for 24 h with 0.5 μg empty pcDNA3.1 vector or pcDNA3.1-TNIP1 vector into which TNIP1 cDNA sequences (OriGene Technologies, Inc., MD, United States) had been inserted. Transfection-grade plasmid was prepared using the E.Z.N.A Endo-free plasmid DNA Maxi Kit (Omega Bio-tek, Inc., GA, United States).

For siRNA-mediated gene silencing, siRNA was delivered by the Santa Cruz siRNA Transfection Reagent (Santa Cruz Biotechnology, Texas, United States) according to the manufacturer's instructions. ON-TARGETplus SMARTpool TNIP1 siRNA or ON-TARGETplus Non-targeting Pool (Dharmacon, Lafayette, United States) constructs were used at a concentration of 10 nM.

A NF-κB luciferase reporter assay was performed using the PathDetect pNF-κB-Luc Cis-Reporter Plasmid (Stratagene, California, USA), pGL4.75[hRluc/CMV] Vector and the Firefly & Renilla Dual Luciferase Assay Kit (Biotium Inc., California, United States), according to the manufacturer's instructions.

### ELISA

*C. acnes*-treated and control HPV-KER cell culture supernatants were collected and the levels of secreted CXCL8 (R&D Systems, Minneapolis, United States), IL-6 and CCL5 (PeproTech EC Ltd., London, United Kingdom) were measured according to the manufactures' instructions.

### RNA isolation, cDNA synthesis and real-time RT-PCR

Total RNA was isolated using TRI-Reagent (Molecular Research Center; Cincinnati, United States). cDNA synthesis was performed using 1 μg RNA with the iScript TM cDNA Synthesis kit (Bio-Rad, Hercules, United States). Changes in mRNA expression were detected by real-time RT-PCR using the Universal Probe Library (Roche, Indiana, United States) or the TaqMan Gene expression Assay (Thermo Scientific, Rockford, United States). Supplementary Table 1 lists the PCR protocols and primer sequences used. All data were normalized to the 18S rRNA using the ΔΔC_t_ method and compared to the time-matched untreated control samples.

### Protein isolation and western blot analysis

For the preparation of whole cell lysates, samples were collected and lysed in lysis buffer containing 20 mM 4-(2-hydroxyethyl)-1-piperazineethanesulfonic acid, 150 mM KCl, 1 mM MgCl_2_, 1 mM DTT, 5% Triton X-100, 10% glycerol, 0.1% NP-40, 1% Protease Inhibitor Cocktail, phenylmethylsulfonyl fluoride and 0.5% sodium dodecyl sulfate (SDS) (all from Sigma Aldrich, St. Louis, MO, United States). Protein concentrations were measured with the BCA Protein Assay Kit (Thermo Scientific, Rockford, United States). Samples (50 μg) were separated on a 7.5% SDS polyacrylamide-gel electrophoresis and transferred to nitrocellulose membrane (Bio-Rad, Hercules, United States), blocked in Tris-buffered saline (TBS) containing 5% Blotting-Grade Blocker (Bio-Rad, Hercules, United States). Membranes were incubated over-night at 4°C with primary anti-TNIP1 antibody diluted 1:500 and anti-actin (Sigma Aldrich, St. Louis, MO, United States) diluted 1:1,000. Subsequently, membranes were incubated for 2 h at room temperature with a horseradish peroxidase-conjugated anti-rabbit IgG (Santa Cruz Biotechnology, Texas, United States) secondary antibody diluted 1:2,000. Proteins were visualized with luminol (Bio-Rad, Hercules, United States) using a Omega Lum™ G Imaging System (Gel Company, CA, United States).

### Fluorescence microscopic analysis

HPV-KER cells were grown on glass sides, fixed with 2% paraformaldehyde (PFA) for 5 min, permeabilized with 0.1% Triton X, 2% PFA-containing phosphate-buffered saline (PBS), and blocked for 2 h at room temperature with PBS containing 1% bovine serum albumin (BSA), 0.05% Triton X 100, and 10% goat serum. Cells were stained overnight at 4°C with anti-human TNIP1 antibody (Sigma Aldrich, St. Louis, MO, United States) or rabbit IgG for isotype control. As a secondary antibody, Alexa Fluor 488 conjugated anti-rabbit IgG, was used for 2 h. Filamentous actin was stained by Alexa Fluor 546® phalloidin (Life Technologies, Carlsbad, United States) diluted 1:100 in PBS containing 1% BSA for 20 min. Nuclei were stained for 10 min with 4′,6-diamidino-2-phenylindole (DAPI) diluted 1:500.

Frozen sections of *ex vivo* skin models were pre-incubated with PBS for 5 min and fixed and permeabilized with Foxp3 staining buffer set (Thermo Scientific, Rockford, United States) and blocked for 1 h at room temperature with TBS containing 1% BSA and 1% normal goat serum. Cells were stained for 1 h with anti-human TNIP1 antibody or rabbit IgG for isotype control. As a secondary antibody, Alexa Fluor 546 conjugated anti-rabbit IgG (Thermo Scientific, Rockford, United States), was used for 1 h at room temperature. Nuclei were stained for 6 min with DAPI diluted 1:100.

### Statistical analysis

Unless otherwise noted, all data are presented as mean ± standard error of the mean (SEM) for three independent experiments. For real-time RT-PCR analyzes and enzyme-linked immunosorbent assay (ELISA), each treatment was performed at least in triplicate; for western blot and fluorescence microscopic analysis each treatment was performed once in every independent experiment. Data were compared using paired, two-sample *t*-test with False Discovery Rate (FDR) correction. A probability value of less than 0.05 was considered significant.

## Results

### TNIP1 is expressed in keratinocytes and its expression increases in response to the presence of *C. acnes*

Initially, we aimed to analyse changes in TNIP1 expression in *in vitro* cultured keratinocytes after treatment with *C. acnes*. For this purpose, NHEK or HPV-KER cells were treated with the bacterium. Keratinocyte to *C. acnes* ratios were determined in extensive preliminary studies. We chose conditions which induced relatively fast and reproducible cellular responses, but did not induce cell death in the time course of our studies (23).

**Figure 1:**
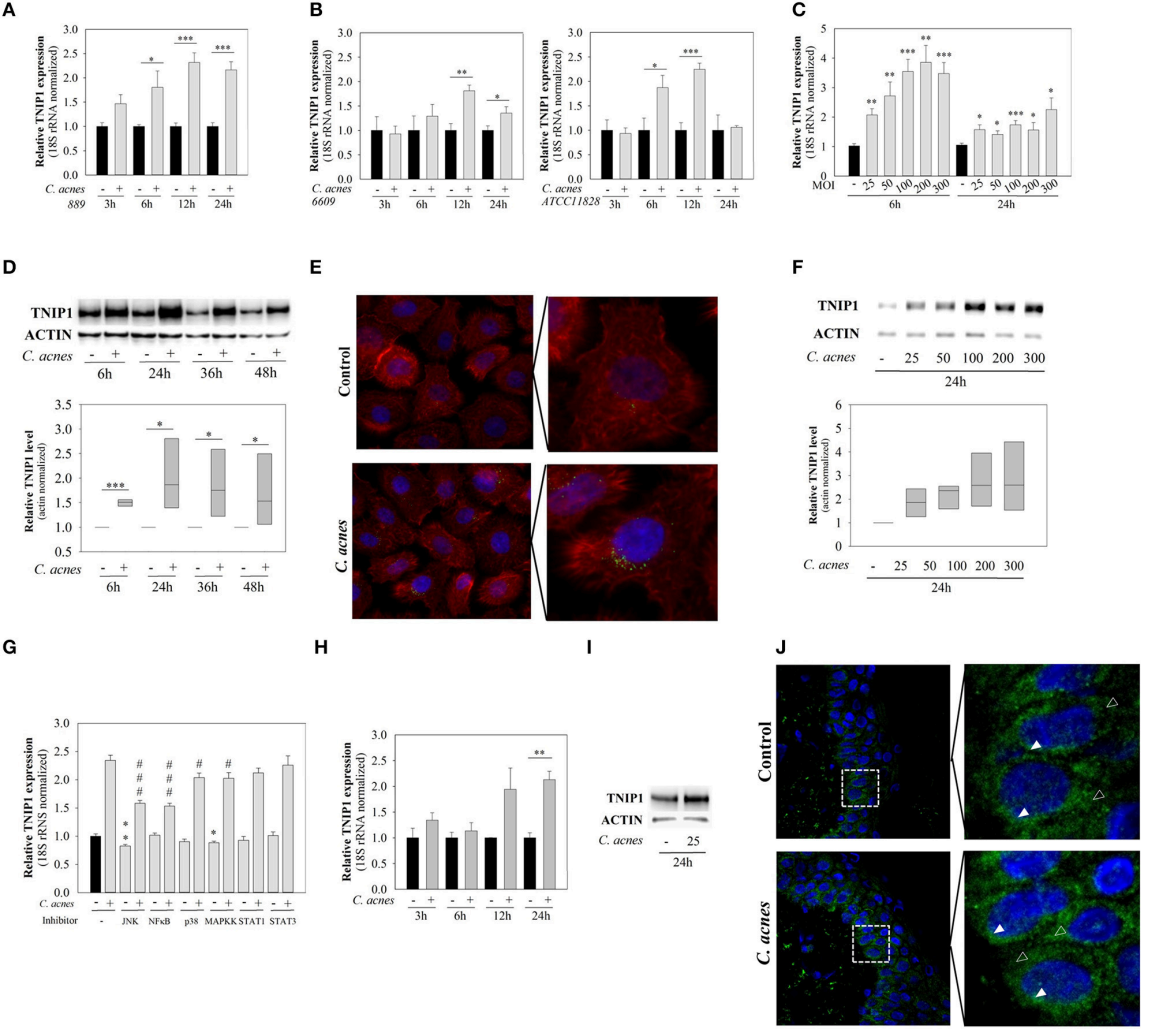
mRNA and protein expression of TNIP1 increases in response to *C. acnes* treatment. HPV-KER cells **(A–G)**, NHEK cells **(H,I)** and an *ex vivo* skin model **(J)** were treated with *C. acnes* 889 at a MOI of 100, unless otherwise indicated, and changes in mRNA and protein levels were analyzed. **(A,B,C,H)** Changes in TNIP1 mRNA expression were analyzed by real-time RT-PCR; data were normalized to 18S rRNA using the ΔΔC_t_ method and compared to the time-match untreated control values. Error bars are SEM. Statistical analyzes: paired, two-sample *t*-test with FDR correction: ^*^p < 0.05, ^**^p < 0.01, ^***^p < 0.001. **(G)** HPV-KER cells were plated and pretreated with selective inhibitors for JNK, NF-κB, p38, MAPKK, STAT1 and STAT3 or, as a control, DMSO for 1 h. *C. acnes* challenge was performed for 12 h and samples were collected for subsequent mRNA analyzes by real-time RT-PCR. All data were normalized to the 18S rRNA using the ΔΔC_t_ method and compared to DMSO-treated control samples. Statistical analyzes: paired, two-sample *t*-test with FDR correction, basal TNIP1 expression ^*^p < 0.05, ^**^p < 0.01 or *C. acnes*-induced TNIP1 expression, #*p* < 0,05, ###*p* < 0.001. were compared. **(D,F)** TNIP1 protein was detected with western blot analysis and quantitated using an Image Pro Plus, where all data were normalized to actin; a representative blot is shown. Statistical analyzes: paired, two-sample *t*-test: ^*^p < 0.05. **(E)** After 24 h *C. acnes* treatment, HPV-KER cells were visualized with immunofluorescence staining for TNIP1 (green), Phalloidin (red) and DAPI (blue). **(I)** NHEK cells were treated with *C. acnes* and TNIP1 protein was detected with western blot analyzes (a representative experiment). **(J)** OSMs were treated with 3 × 10^7^ bacterium for 24 h, and cells were visualized with immunofluorescence staining for TNIP1 (green) and DAPI (blue), where empty arrows indicate cytoplasmic location and filled arrows indicated perinuclear localization.

We found that TNIP1 is expressed in HPV-KER cells and that mRNA expression rapidly and significantly increased after *C. acnes* 889 treatment (MOI = 100), reaching the maximum at 12–24 h (Figure 1A). These findings suggest that these cells recognize the presence of *C. acnes* bacterium.

It has been suggested that various *C. acnes* strains may have different effects on the cellular and molecular properties of human keratinocytes (10). We applied *C. acnes* strains (MOI = 100) belonging to various phylogenetic groups within the species (*C. acnes* 889, 6609, ATCC 11828, Group IA, IB, and II.) and compared the effects. No strain-specific differences were observed: all of the *C. acnes* strains used induced similar changes in mRNA expression in HPV-KER cells (Figure 1B). Subsequently, only the *C. acnes* 889 strain was used in further experiments.

We also found, that bacterium-induced changes in TNIP1 expression were dose-dependent: the abundance of mRNA increased in parallel with increasing *C. acnes* 889 bacterial doses (Figure 1C).

Next, we analyzed TNIP1 protein levels in HPV-KER cells using western blot analysis and immunocytochemistry. Elevated TNIP1 levels were detected in the 6 h samples and remained high during the time-course of the experiment (Figure 1D). Immunocytochemical staining of TNIP1 resulted in the presence of immunofluorescent dots, occurring mostly in the perinuclear region of HPV-KER cells. The number of labeled dots increased after 24 h of *C. acnes* treatment (MOI = 100) (Figure 1E). Similarly to the mRNA levels, the abundance of TNIP1 protein increased in parallel with increasing *C. acnes* 889 doses (Figure 1F).

To identify which signaling pathways are involved in the regulation of constitutive and *C. acnes*-induced TNIP1 expression levels in keratinocytes, we treated HPV-KER cells with specific inhibitors of well-known representatives (JNK, NF-κB, p38, MAPKK, STAT1, and STAT3) of different signaling pathways before the *C. acnes* treatment and analyzed subsequent changes in TNIP1 mRNA expression by real-time RT-PCR. We found that constitutive TNIP1 expression was significantly decreased when JNK and MAPKK were inhibited. Furthermore, *C. acnes*-induced changes in TNIP1 expression diminished in response to inhibition of JNK and MAPKK as well as of NF-κB and p38. In contrast, no effect was observed with STAT1 and STAT3 inhibition (Figure 1G).

To confirm that *C. acnes*-induced TNIP1 expression changes were not specific to the HPV-KER cell line, we repeated the experiments using NHEK cells and found similar results. TNIP1 mRNA levels increased in response to *C. acnes* treatment (Figure 1H), and increased protein levels were also noted in NHEK cell cultures 24 h after treatment (Figure 1I).

To confirm that the observed results were not specific to keratinocyte monolayer cultures, we also repeated the experiments using an *ex vivo*, OSMs. Punch biopsies of full thickness skin samples from healthy donors were cultured at the air–liquid interphase and *C. acnes* 889 was applied to the top (epidermal side) of the samples. We found that TNIP1 protein was expressed throughout the epidermis, and that slightly higher levels were detected in the less differentiated, basal layers. Within the keratinocytes, localization was primarily cytoplasmic and perinuclear. Elevated TNIP1-staining levels were found 24 h after *C. acnes* treatment (Figure 1J).

### TNIP1 down-regulates both constitutive and *C. acnes*-induced inflammatory cytokines and chemokines expression

To analyze the role of TNIP1 in the regulation of *C. acnes*-induced inflammatory events, we experimentally modified endogenous TNIP1 levels with cDNA-based transient overexpression or siRNA-mediated silencing. We monitored the expression of selected pro-inflammatory cytokines and chemokines that are known downstream targets of the TLR signaling pathway, as well as the promoter activity of the NF-κB transcription factor, using real-time RT-PCR, ELISA analysis and a luciferase- reporter assay.

**Figure 2:**
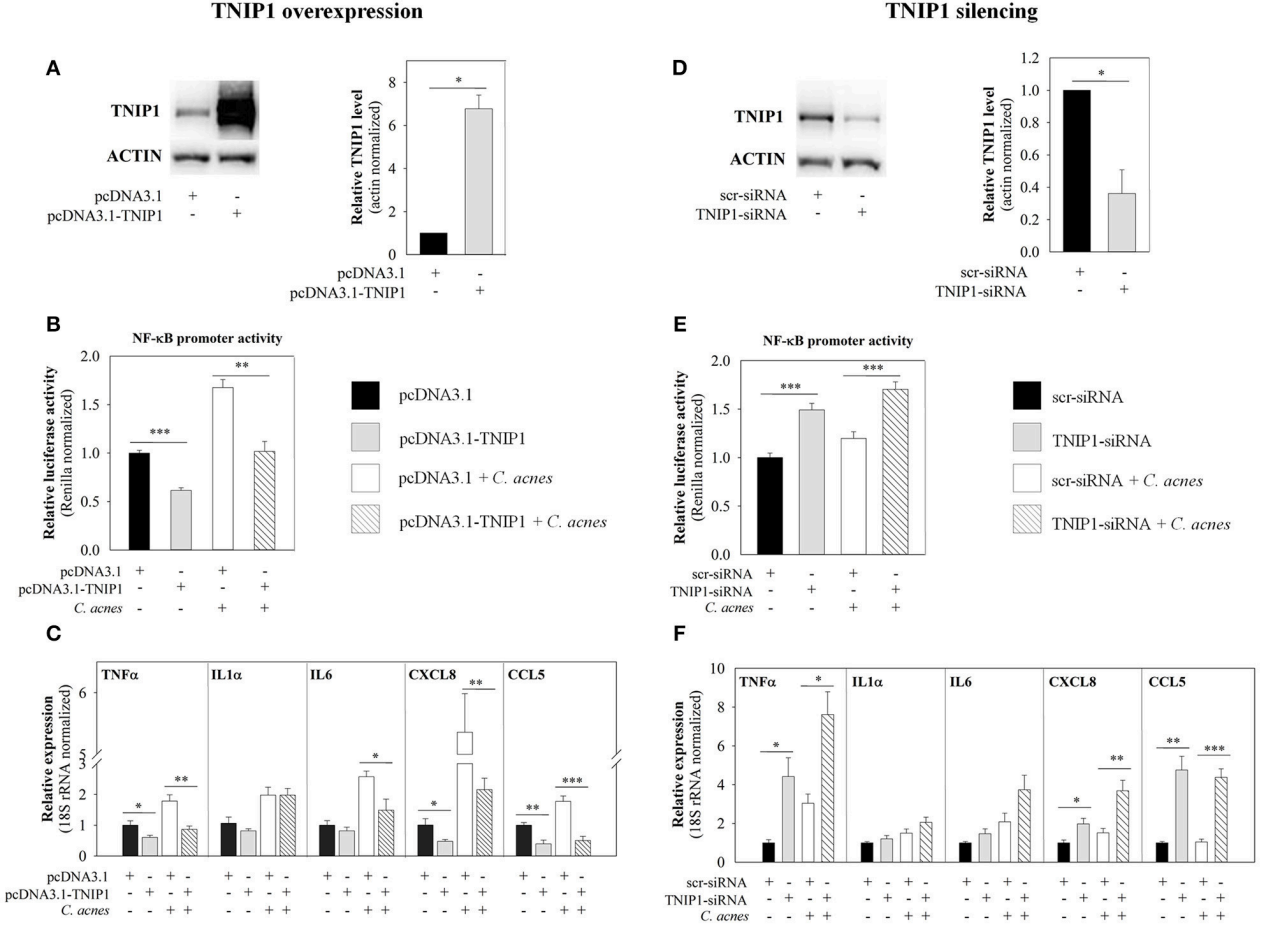
Effects of TNIP1 levels on the downstream targets of the TLR signaling pathway. After plating HPV-KER cells, TNIP1 overexpression (24 h) (left panel) or siRNA-mediated silencing (48 h) (right panel) was performed. Subsequently, cells were treated with *C. acnes* (MOI = 100) for 6 h. **(A,D)** TNIP1 protein levels were analyzed by western blot after overexpression or silencing and quantitated using Image Pro Plus, where all data were normalized to actin; a representative blot is shown. Error bars are SD. **(B,E)** NF-κB promoter activities were measured with a luciferase reporter assay; data were normalized to signal from a vector constitutively expressing Renilla luciferase and compared to time-matched samples transfected with vectors that were empty (pcDNA3.1) or contained scr-siRNA. **(C,F)** mRNA levels of TNFα, IL-6, CXCL8 and CCL5 were analyzed by real-time RT-PCR; data were normalized to 18S rRNA using the ΔΔC_t_ method and compared to time-matched samples transfected with vectors that were empty (pcDNA3.1) or contained scr-siRNA. Error bars are SEM. Statistical analyzes: paired, two-sample *t*-test: ^*^p < 0.05, ^**^p < 0.01, ^***^p < 0.001.

**Figure 3:**
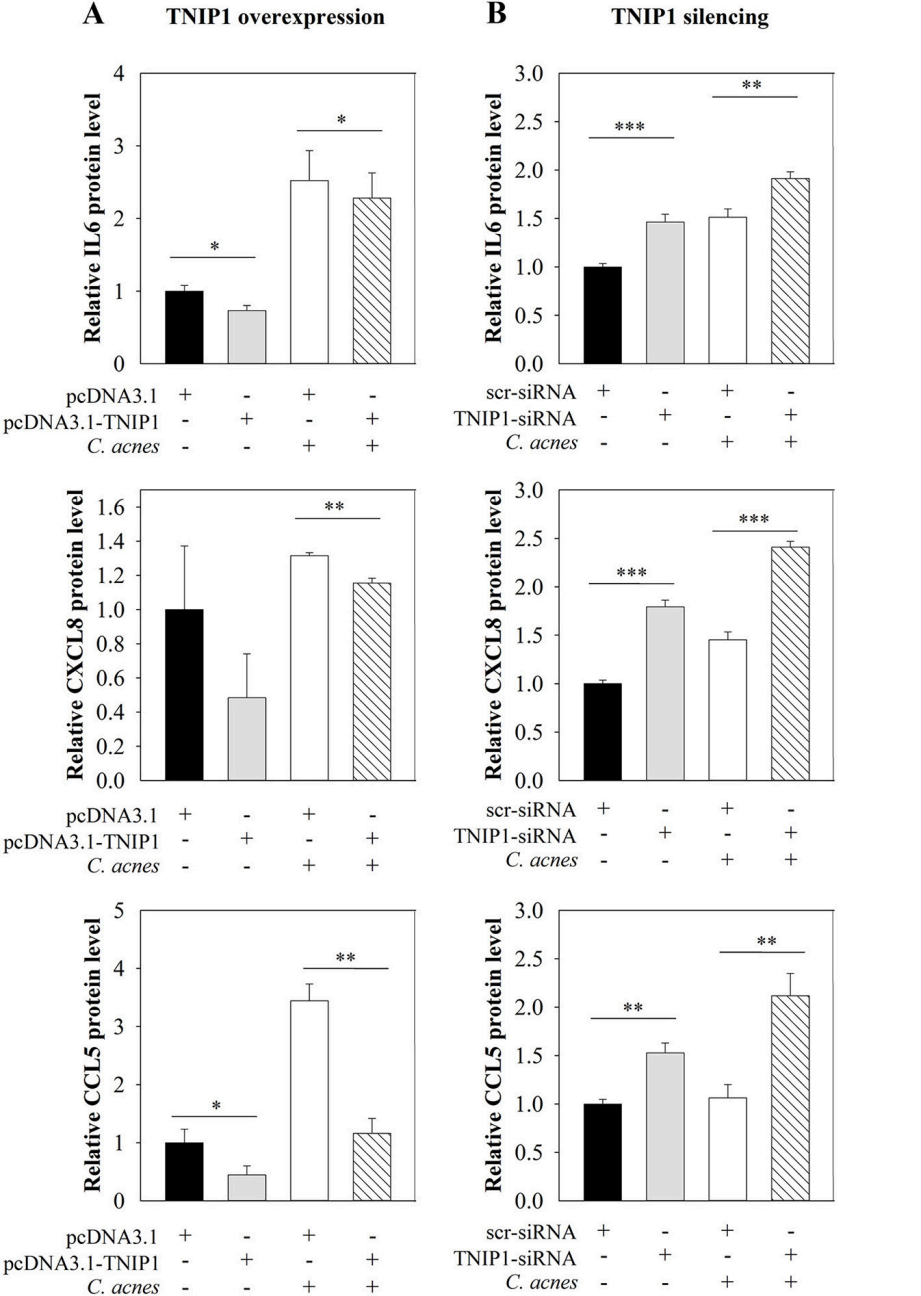
Effects of TNIP1 levels on secretion of pro-inflammatory cytokine and chemokine. After plating HPV-KER cells, **(A)** TNIP1 overexpression (24 h) or **(B)** siRNA-mediated silencing (48 h) was performed. Subsequently, cells were treated with *C. acnes* (MOI = 100) for 24 h. Secreted cytokine and chemokine levels were measured by ELISA and relative protein levels are presented. Data were normalized to time-matched samples transfected with a vector that was empty (pcDNA3.1) or contained scr-siRNA. Values of the measured protein levels were the following: IL-6: 200–1,300 pg/ml, CXCL8: 200–2,800 pg/ml, CCL5: 50–400 pg/ml. Error bars are SEM. Statistical analyzes: paired, two-sample *t*-test: ^*^p < 0.05, ^**^p < 0.01, ^***^p < 0.001.

cDNA-based transient overexpression resulted in markedly increased TNIP1 protein levels in HPV-KER cells (Figure 2A). As a consequence, significantly decreased constitutive and *C. acnes*-induced NF-κB promoter activities were measured in the subsequent luciferase reporter assay (Figure 2B). mRNA expression of constitutive and *C. acnes*-induced TNFα, CXCL8 and CCL5 also decreased compared to the empty-vector transfected samples. In addition, bacterium-induced mRNA levels of IL6 decreased, whereas IL1α levels were not affected (Figure 2C). Overexpression of TNIP1 also decreased IL-6, CXCL8, and CCL5 protein secretion (Figure 3A).

In contrast, siRNA-mediated silencing of TNIP1 led to markedly decreased TNIP1 protein levels (Figure 2D). As a consequence, significantly increased constitutive and bacterial induced NF-κB promoter activities were found in a luciferase reporter assay (Figure 2E). Constitutive and *C. acnes*-induced expression of TNFα, CXCL8, and CCL5 mRNA markedly increased, whereas IL1α and IL6 expression increased moderately in response to TNIP1 silencing (Figure 2F). Constitutive and bacterium-induced secretion of CXCL8, IL6, and CCL5 were also elevated in TNIP1 silenced HPV-KER cells (Figure 3B).

### ATRA induces TNIP1 expression and affects the levels of downstream targets of the TLR signaling pathway

The TNIP1 promoter contains retinoic acid response elements (RARE) and retinoid-acid-receptor binding sites. These elements are involved in the induction of *TNIP1* expression by retinoic acid under permissive epigenetic conditions in different cell lines (19). Since ATRA is an effective drug used for acne treatment, we examined whether this compound is capable of regulating TNIP1 and, thus, the expression of downstream targets of the TLR signaling pathway in keratinocytes.

**Figure 4:**
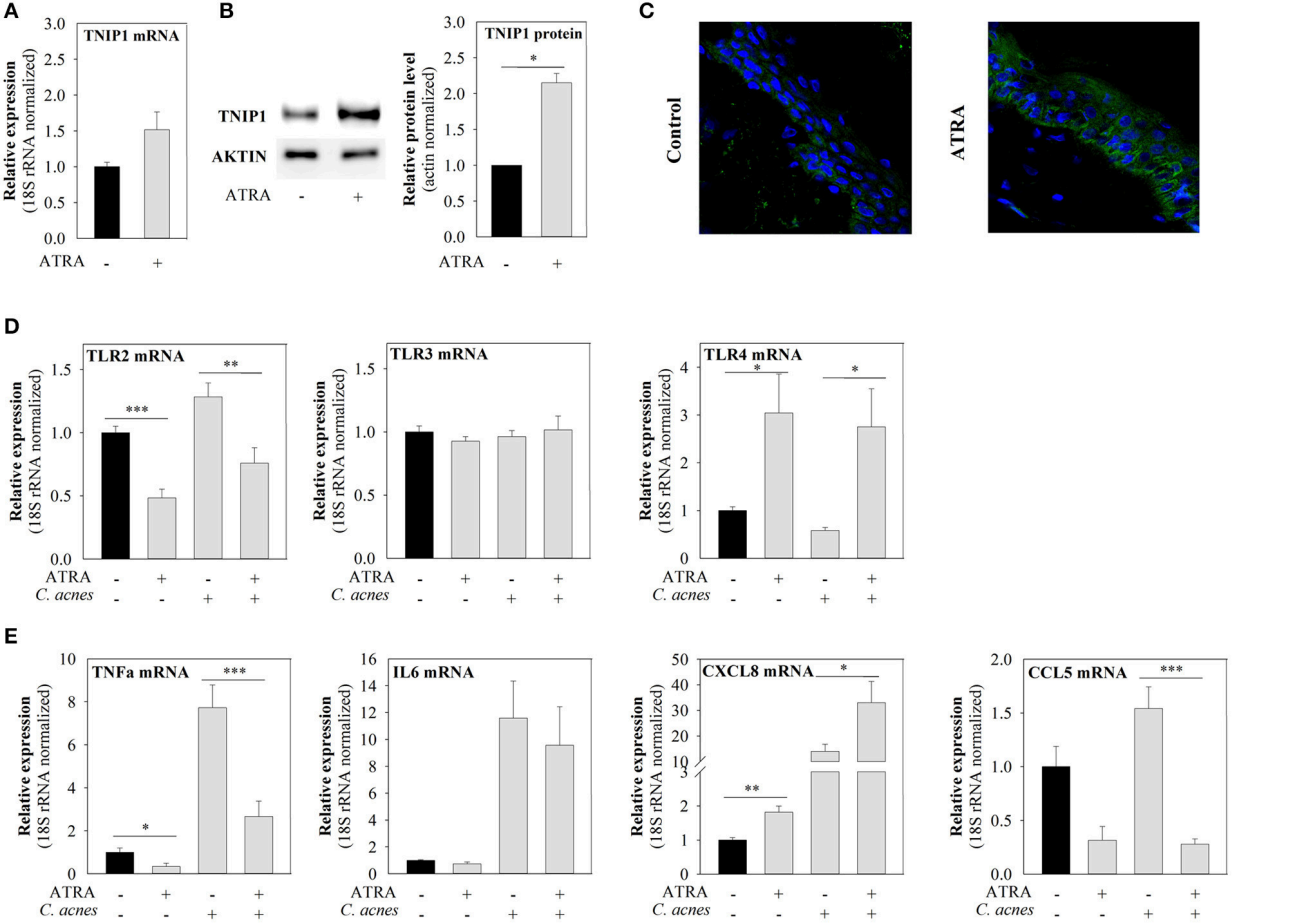
ATRA induces TNIP1 expression and affects the expression of TLRs and downstream targets of the TLR signaling pathways. HPV-KER cells **(A,B)** were treated with 10^−6^ M ATRA for 48 h or *ex vivo* skin **(C)** with 1.5 × 10^−6^ M for 24 h, and TNIP1 mRNA and protein levels were analyzed. **(D,E)** HPV-KER cells were treated with 10^−6^ M ATRA for 48 h and challenged with *C. acnes* (MOI = 100) for 6 h. mRNA expression of TLRs and selected pro-inflammatory molecules was analyzed. mRNA expression changes were detected by real-time RT-PCR; data were normalized to the 18S rRNA using the ΔΔC_t_ method and compared to the untreated control values. Error bars are SEM. **(B)** TNIP1 protein was detected by western blot analysis and quantitated using an Image Pro Plus; a representative blot is shown, or **(C)** was visualized with immunofluorescence staining for TNIP1 (green) and DAPI (blue). Statistical analyzes: paired, two-sample *t*-test: ^*^p < 0.05, ^**^p < 0.01, ^***^p < 0.001.

We observed that ATRA treatment led to slightly elevated TNIP1 mRNA levels, although this effect was not statistically significant (Figure 4A), and to significantly increased TNIP1 protein expression (Figure 4B). In addition, constitutive and *C. acnes*-induced mRNA expression of TLR-2 and the pro-inflammatory TNFα and CCL5 decreased upon ATRA treatment. In contrast, both constitutive and *C. acnes*-induced TLR-4 and CXCL8 mRNA expression levels increased, whereas TLR3 and IL-6 mRNA levels were not affected by the drug (Figures 4D,E).

To confirm that the effects of ATRA on TNIP1 expression were not specific to the HPV-KER cell line, we also applied the drug to the upper, epidermal part of OSMs. We found that TNIP1 protein expression levels had increased in all epidermal layers 24 h after ATRA treatment (Figure 4C), in a manner similar to observed for immortalized keratinocytes.

## Discussion

Human skin harbors a special microbiota which protects against pathogens, helps to maintain the proper functions of the skin and also serves as an immunological barrier, functions that have also been proposed for the gut microbiota (25). These skin functions are partly achieved by keeping the immune system at a basal activated state. Low levels of TLR activation may contribute to the maintenance of basal expression from genes with important roles for the immunological barrier (e.g., inducible antimicrobial peptides, such as hBD2) (26).

Under healthy conditions, these events do not lead to inflammatory processes. However, under certain conditions, the commensal bacteria are involved in inflammatory skin diseases, such as acne (27, 28). The mechanisms underlying this duality – the protective and pathogenic aspects of the continual presence of the skin microbiota – are still unclear (1, 25) *C. acnes*, a dominant member of the human skin microbiome, is recognized by TLR2 and TLR4 in human epidermal keratinocytes. This bacterium can induce innate immune and inflammatory events, such as production of inflammatory cytokines, chemokines, antimicrobial peptides and other inflammatory mediators, as well as autophagy in keratinocytes, and, through this induction, plays a role in the pathogenesis of acne vulgaris (7, 8, 27, 29). However, at the beginning of early adolescence this bacterium is overwhelming dominant in the skin microbiota under healthy conditions, yet does not induce macroscopically detectable inflammation (3, 5). These observations suggest that one or more mechanisms regulate *C. acnes*-induced immune activation.

In the past decades, several negative regulators of TLR and the NF-κB signaling pathway have been identified. The regulatory functions of these molecules play important roles in the protection against exaggerated immune activation. Since their discovery, an increasing number of publications has reported correlation between genetic variations of these proteins and altered expression and/or dysfunctional proteins as well as with diseases associated with inflammation, such as psoriasis, systemic lupus erythematosus and cancer (13, 16, 30). Taken together, these results suggest that proper regulation and function of these factors are indispensable for the maintenance of health. The role of these molecules in the assuagement of microbiome-induced immune activation has not yet been investigated.

In our study, we analyzed TNIP1, a regulator of NF-κB signaling pathways, and found that it is a possible negative regulator of *C. acnes*-induced inflammatory events in HPV-KER cells. TNIP1 is expressed in cultured keratinocytes and mRNA and protein expression increased in response to *C. acnes* treatment; the extent of activation was dependent on the bacterial dose. We confirmed that these changes were not specific for the HPV-KER cell line, as similar results were obtained using NHEK cells. In *ex vivo* OSMs we showed that TNIP1 is expressed in all epidermal layers, which was consistent with previous studies (17). We detected mostly cytoplasmic and perinuclear staining within the keratinocytes, in contrast to previous studies, where cytoplasmic and also intensive nuclear staining of TNIP1 was observed in HaCaT cells (17, 31). In response to *C. acnes* treatment, elevated TNIP1 levels were found in the epidermis. These results are in agreement with our findings using NHEK and HPV-KER cells: TNIP1 expression changes in keratinocytes in response to the presence of the bacterium. These findings show that, in addition to pro-inflammatory mediators, *C. acnes* also induces the expression of anti-inflammatory factors, possibly to avoid excessive inflammation and immune activation. Our results strongly suggest that TNIP1 may be one of the negative regulators of *C. acnes*-induced molecular events.

Earlier studies revealed differences in *C. acnes* strain-composition from healthy individuals and acne patients (32). It has been suggested that strains belonging to various phylogenetic groups within the species differentially affect the cellular and molecular properties of keratinocytes (10). In the present study, we did not observe strain-specific differences in TNIP1 expression in response to *C. acnes*, suggesting that strain-specific activation of keratinocytes are not related to the ability of the bacterium to induce the studied negative regulators.

*In silico* analyzes predicted the presence of putative biding sites for NF-κB, AP-1, SP and C/EBPβ (33, 34) in the *TNIP1* promoter region. Gene expression and ChIP assays have confirmed that NF-κB and SP1 binding sites are active in HeLa cells (20, 18). Our finding that specific JNK, NF-κB, p38 and MAPKK inhibitors decreased the bacterium-induced upregulation of TNIP1 suggest that these signaling pathways may be involved in the regulation of *C. acnes*-induced TNIP1 expression in keratinocytes. Moreover, the intricate pattern of TNIP1 regulation suggests this molecule may have important roles in different processes.

To confirm the negative regulatory role of TNIP1 in *C. acnes*-induced molecular events, we modified its endogenous levels by cDNA-based overexpression and siRNA-mediated silencing and examined the expression of downstream elements of TLR signaling pathways. NF-κB, one of the main mediator of signaling cascades activated in our experiments, is induced upon exposure to bacteria (7, 10). Our results, that TNIP1 overexpression decreased basal and *C. acnes*-induced NF-κB promoter activities and mRNA levels of TNFα, CXCL8 and CCL5 as well as secretion of CXCL8, IL-6 and CCL5 correlate well with findings on other cell types. In HeLa cells, TNIP1 overexpression inhibited constitutive TNFα, IL1α expression and lipopolisaccharide (LPS)-induced activation of NF-κB (15, 35).

In TNIP1-silenced cells, we observed increased constitutive NF-κB promoter activities and elevated pro-inflammatory cytokine and chemokine mRNA and protein levels. TNIP1 silencing also increased *C. acnes*-induced NF-κB promoter activity, mRNA levels and secretion of the mediators mentioned above. These results further support the conclusion that TNIP1 plays a role in the regulation of *C. acnes*-induced events in HPV-KER cells and possibly in the maintenance of homeostatic conditions. In a recent report, the authors have also demonstrated that TNIP1-silenced HaCaT cells were hypersensitive to synthetic TLR3 and TLR2/6 ligands and subsequently increased JNK and p38 phosphorylation and nuclear translocation of NF-κB. They also observed increased levels of secreted IL6 and CXCL8 compared to control cells (21). Overall, these data indicate a negative regulatory role of TNIP1 in TLR signaling events in keratinocytes.

Other reports have also shown the importance of TNIP1 as a gatekeeper in NF-κB, JNK and p38 mediated processes, including the prevention of fetal liver apoptosis in a murine model and TNFα-induced apoptosis in different cell lines (36, 37). In contrast, although TNIP1-deficient mice develop a progressive, lupus-like inflammatory disease, isolated TNIP1-deficient macrophages and dendritic cells showing no differences in pro-inflammatory signaling pathways with respect to IκBα degradation and resynthesis and phosphorylation of different MAPKs (p38, ERK1/2, and JNK1/2) compared to wild type mice upon CpG-DNA or TLR4 (LPS) stimulation (38, 39). These findings suggest that the role of TNIP1 during TLR activation might be cell-type specific and depends on the nature of the stimuli.

Earlier studies found functional RARE elements in the *TNIP1* promoter region, and that TNIP1 was induced by ATRA in HeLa cells under permissive epigenetic conditions, in the presence of Trichostatin A co-treatment (19). Trichostatin A inhibits histone deacetylase I and II and alters gene expression by opening chromatin and allowing transcription factors to bind, thus, promoting transcription of different genes. We applied ATRA, an active form of retinoic acid, to HPV-KER cells and found that TNIP1 mRNA and protein expression levels increased in response to the treatment without the addition of chromatin-modification agents. Furthermore, treatment of OSMs led to similar results throughout the entire epidermis, suggesting that these effects were not specific to HPV-KER cells. It is not currently clear why HeLa cells behaved differently; however, differences in the duration of ATRA exposure or in the responsiveness of the different cells used might be responsible.

Despite the fact that retinoids, including ATRA, are widely used as an effective drug for acne therapy, the exact mechanism of action is not completely understood. Retinoids have been shown to promote cell proliferation, inhibit keratinocyte terminal differentiation, decrease the size of sebaceous glands and, indirectly, reduce the amount of *C. acnes* (40). Studies of the effect of retinoids on innate immunity are limited, and the results are often dependant on the cells used (19, 41–44). In most cases, ATRA application to monocytes and macrophages decreased TLR2 abundance as well as the expression of selected pro-inflammatory mediators; however, the mechanism of these effects remains unclear (43, 45, 46).

In our experiments, ATRA treatment decreased the level of constitutive and *C. acnes*-induced pro-inflammatory mediators TNFα and CCL5, but increased CXCL8 levels. These results are consistent with the findings of others; similar CXCL8 expression changes were observed in NHEK and also in other cell types in response to ATRA (41, 42, 47). In NHEK cells, NF-κB and p38 signaling might contribute to these events (41).

Based on all these data, we conclude that ATRA may regulate TNIP1 expression and, as a consequence, negatively affect *C. acnes*-induced inflammatory events in keratinocytes. Our proposed model may offer a possible, novel mode of retinoid action in acne treatment.

TLR2 expression is increased in the epidermis and monocytes isolated from acne patients compared to healthy controls, and this increase might be a result of *C. acnes*-induced inflammatory events. ATRA treatment decreased TLR2 levels in monocytes isolated from both healthy donors and acne patients following isotretinoin therapy, although TLR4 expression was not affected (7, 45, 46, 48, 49). We found similar changes in expression for TLR2 (decreased) and TLR4 (increased) in HPV-KER cells, suggesting that ATRA has opposite effects on these two receptors in keratinocytes.

We propose that the increased bacterial load in acne-prone follicles is a factor leading to the formation of inflammatory symptoms. Retinoids may attenuate *C. acnes*-induced inflammation by decreasing the sizes of sebaceous glands and sebum secretion and, subsequently, control the *C. acnes* load. In addition, TLR2 levels are also attenuated, preventing the sensing of this Gram-positive bacterium, which leads to deleterious inflammation. By decreasing the expression of TLR2, an important gatekeeper of the skin, the risk of opportunistic infections is increased. To modulate this effect, expression of another gatekeeper, TLR4 is elevated. Thus, multiple levels of sensing and signal transduction may be available in the defense against potentially harmful microbial invaders. The fact that opportunistic bacterial and fungal infections are rare in the lesional skin of acne patients is consistent with our hypothesis.

TNIP1 may regulate *C. acnes*-induced signaling events through the establishment of a negative-regulatory feedback loop controlling NF-κB activity in keratinocytes. Other signaling pathways, such as nuclear-receptor signaling cascades that are activated by retinoids, may also affect TNIP1 levels, which in turn can modify the outcome of the induced processes. Based on our results, TNIP1 may function as a negative regulator in keratinocytes that controls bacterium-induced inflammatory events, playing an important role in maintaining the homeostasis between skin cells and the skin microbiome. Development of novel, well-tolerated, TNIP1-specific acne therapeutic modalities could potentially reduce inflammation without the harmful side effects of currently available treatment options (antibiotic, retinoid or hormonal formulation usage).

## Author contributions

LE and KS designed the experiments. LE, BB, RB, and GT performed the experiments. EU cultured and provided *C. acnes* strains, which were used in our experiments. LE, LK, and KS performed data analysis and wrote the manuscript. LK and KS were involved in the coordination of the study.

### Conflict of interest statement

The authors declare that the research was conducted in the absence of any commercial or financial relationships that could be construed as a potential conflict of interest.
